# Characterization of Dental Pulp Stem Cell Responses to Functional Biomaterials Including Mineralized Trioxide Aggregates

**DOI:** 10.3390/jfb12010015

**Published:** 2021-02-24

**Authors:** Sejin Bae, Bueonguk Kang, Hyungbin Lee, Harrison Luu, Eric Mullins, Karl Kingsley

**Affiliations:** 1 Department of Clinical Sciences, School of Dental Medicine, University of Nevada, 1700 W. Charleston, Las Vegas, NV 89106, USA; bae4@unlv.nevada.edu (S.B.); kangb5@unlv.nevada.edu (B.K.); lee64@unlv.nevada.edu (H.L.); harrison.luu@sdm.unlv.edu (H.L.); mullins4@unlv.nevada.edu (E.M.); 2 Department of Biomedical Sciences, School of Dental Medicine, University of Nevada, 1001 Shadow Lane, Las Vegas, NV 89106, USA

**Keywords:** dental pulp, stem cell, differentiation, biotechnology, bioengineering

## Abstract

Introduction: Many studies in stem cell biology have demonstrated that dental pulp stem cells (DPSC) may be highly proliferative and capable of pluripotent differentiation into many different tissue types. Recent advances in stem cell research have outlined methods for directing in vitro or in vivo growth, viability, and proliferation, as well as differentiation of DPSC—although much remains to be discovered. Based upon this information, the primary objective of this study was to understand the functional biomaterials needed to more effectively direct DPSC viability, growth, and proliferation. Methods: Using an approved protocol, previously collected and isolated samples of DPSC from an existing repository were used. Previously established stem cell biomarkers (Sox-2, Oct-4, NANOG) from each isolate were correlated with their proliferation rates or doubling times to categorize them into rapid, intermediate, or slow-dividing multipotent DPSC. Growth factors and other functional dental biomaterials were subsequently tested to evaluate DPSC responses in proliferation, viability, and morphology. Results: Differential responses were observed among DPSC isolates to growth factors, including vascular endothelial growth factor (VEGF) and bone morphogenic protein (BMP-2), and functional biomaterials such as mineralized trioxide aggregates (MTA). The responsiveness of DPSC isolates did not correlate with any single factor but rather with a combination of proliferation rate and biomarker expression. Conclusions: These data strongly suggest that some, but not all, DPSC isolates are capable of a robust and significant in vitro response to differentiation stimuli, although this response is not universal. Although some biomarkers and phenotypes that distinguish and characterize these DPSC isolates may facilitate the ability to predict growth, viability, and differentiation potential, more research is needed to determine the other intrinsic and extrinsic factors that may contribute to and modulate these DPSC responses to these functional biomaterials for biotechnology and bioengineering applications.

## 1. Introduction

Many studies in stem cell biology have demonstrated that dental pulp stem cells (DPSC) may be highly proliferative and capable of pluripotent differentiation into many different tissue types [1,2]. For example, evidence has shown that DPSC may be induced into dentinogenesis, osteogenesis, chondrogenesis, adipogenesis, and neurogenesis [3,4]. However, much remains to be discovered regarding the materials and methods used to stimulate growth, increase viability, and promote differentiation, as well as the biomarkers and properties of the DPSC that determine their differentiation potential [5,6,7]. 

Recent advances in stem cell research have outlined the characteristics of DPSC that may be useful in directing in vitro or in vivo differentiation [8,9]. For example, stem cells from human exfoliated deciduous teeth (SHED) and stem cells from the apical papilla (SCAP) may express vastly different biomarkers and differentiation potentials [10,11]. Moreover, the methods used to isolate and store these DPSC may also influence the capacity and potential of these DPSC isolates for any therapeutic or bioengineering purposes [12,13]. For example, some studies have revealed that the isolation of DPSC using enzymatic dissociation may result in heterogenous DPSC isolate populations of more rapidly dividing cells, while the direct outgrowth technique from tissue explants may be more likely to give rise to more homogenous isolates with more limited differentiation potential [14,15]. In addition to the isolation methods used, the protocols and techniques used in cryopreservation may also influence the potential viability and long-term survival following cryopreservation—with research studies suggesting that both lower passage number and decreased concentration of dimethyl sulfoxide (DMSO) may significantly improve both survival and viability outcomes among DPSC isolates [13,15].

Other research has outlined potential methods used to direct in vitro or in vivo differentiation [16,17]. Some of these efforts have involved the use of defined growth factors and growth media supplements, including the use of transforming growth factor (TGF), platelet-derived growth factor (PDGF), fibroblast growth factor (FGF), and nerve growth factor (NGF) to induce DPSC differentiation [18,19,20]. However, the most commonly cited and effective growth factors added to cell culture media that appear to modulate differentiation across many types of DPSC include bone morphogenic protein (BMP) and vascular endothelial growth factor (VEGF)—which have been demonstrated to increase proliferation as well as viability in these studies [16,17,18,19,20]. Other researchers have explored the potential bioactivity of dental materials, such as endodontic biomaterials including mineralized trioxide aggregates (MTA) on DPSC [21,22,23]. However, the vast majority of these studies to date have not included an extensive characterization of these DPSC isolates, including proliferation rate or doubling time, biomarker expression, and analysis of pluripotent differentiation markers—which may, in fact, be critical to understanding which factors may modulate specific responses among differing DPSC isolates to induce differentiation.

Based upon this information, the primary objective of this study was to understand the biology and biotechnology needed to more effectively modulate DPSC phenotypes, including growth, viability, and proliferation, using BMP, VEGF, and MTA on well-characterized DPSC isolates.

## 2. Methods

### 2.1. Study Approval

Previously collected and isolated samples of DPSC from an existing repository were used. Due to the use of previously collected, non-identifiable samples, this protocol was granted exemption from Human Subjects review from the University of Nevada, Las Vegas (UNLV) Office for the Protection of Research Subjects (OPRS) #763012-1 titled “Retrospective analysis of dental pulp stem cells (DPSC) from the University of Nevada, Las Vegas (UNLV) School of Dental Medicine (SDM) pediatric and clinical population” on 3 August 2015.

The original protocol for the collection and storage of DPSC was reviewed and approved by the Institutional Review Board (IRB) and UNLV OPRS under OPRS#0907-3148 “Isolation of Non-Embryonic Stem Cells from Dental Pulp” [24] on 5 February 2010.

### 2.2. Cell Culture

DPSC isolates frozen in 10% dimethyl sulfoxide (DMSO)-containing media were thawed and cultured in Dulbecco’s Modified Eagle’s Medium (DMEM) High Glucose with the addition of 10% heat-inactivated fetal bovine serum (FBS) and 1% penicillin streptomycin (Pen-Strep) at 37 °C in a humidified tissue culture incubator with 5% CO_2_. DMSO (CAS 67-68-5), DMEM (MT15017CV), FBS (MT35011CV), and Pen-Strep (MT30001Cl) were obtained from Fisher Scientific (Fair Lawn, NJ, USA). Cells were passaged 1:2 and doubling time was noted for each DPSC isolate. Cell cultures achieving confluence within 1–2 days were categorized as rapid doubling time or rDT between 1 and 2 days, with intermediate doubling time or iDT noted between 5 and 6 days, and slow doubling time or sDT categorized as 10–12 days. 

### 2.3. RNA Isolation

RNA was isolated from each DPSC using the ABgene Total RNA isolation reagent kit and protocol recommended by ThermoFisher (Fisher Scientific; Fair Lawn, NJ, USA), as previously described [24,25]. All samples were screened for purity using the NanoDrop spectrophotometer (Fisher Scientific; Fair Lawn, NJ, USA) absorbance readings at A260 and A280 nm. The ratio of A260:A280 provides an approximation of nucleic acid purity. All samples were required to demonstrate an A260:A280 ratio above 1.65, which was suitable for polymerase chain reaction (PCR) screening. Quantification of RNA samples was concomitantly collected to determine the minimal PCR processing requirement of 1 ng/μL.

### 2.4. PCR Screening 

RNA obtained from each DPSC isolate was subsequently screened for the presence of CD90 and CD105 and the absence of CD45, according to the International Society for Cellular Therapy (ISCT) criteria for stem cells [26] using RT-PCR on 1 ug of total RNA with the ABgene Reverse-iT One-Step RT-PCR kit (ThermoFisher Scientific; Fair Lawn, NJ) and a Mastercycler gradient thermocycler (Eppendorf; Hamburg, Germany) that included an initial reverse transcription at 47 °C for 30 min, followed by 30, cycles of PCR with annealing for 30 sec at the appropriate temperature for each primer set, and final extension at 60 °C for one minute, as previously described [24,25,26]. The PCR positive cellular RNA control was glyceraldehyde 3-phosphate dehydrogenase (GAPDH). In addition, three additional mesenchymal stem cell (MSC) markers (Sox-2, Oct-4, and NANOG) were also used with the following primers, synthesized by SeqWright (Fisher Scientific; Fair Lawn, NJ, USA).

CD90 forward: 5′-ATGAACCTGGCCATCAGCA-3′; 19 nt, 53% GC, Tm: 67 °C

CD90 reverse: 5′-GTGTGCTCAGGCACCCC-3′; 17 nt, 71% GC, Tm: 70 °C

Optimal Tm: 68 °C

CD105 forward: 5′-CCACTAGCCAGGTCTCGAAG-3′; 20 nt, 60% GC, Tm: 67 °C

CD105 reverse: 5′-GATGCAGGAAGACACTGCTG-3′; 20 nt, 55% GC, Tm: 66 °C

Optimal Tm: 67 °C

CD45 forward: 5′CATATTTATTTTGTCCTTCTCCCA-3′; 24 nt, 33% GC, Tm: 60 °C

CD45 reverse: 5′-GAAAGTTTCCACGAACGG-3′; 18 nt, 50% GC, Tm: 61 °C

Optimal Tm: 61 °C

Oct-4 forward: 5′-TGGAGAAGGAGAAGCTGGAGCAAAA-3′; 25 nt: 48% GC; Tm 70 °C Oct4 reverse: 5′-GGCAGATGGTCGTTTGGCTGAATA-3′; 24 nt; 50% GC; Tm 70 °C

Optimal Tm: 71 °C

Sox2 forward: 5′-ATGGGCTCTGTGGTCAAGTC-3′; 20 nt: 55% GC; Tm 67 °C 

Sox2 reverse: 5′-CCCTCCCAATTCCCTTGTAT-5′; 20 nt; 50% GC; Tm 64 °C 

Optimal Tm: 65 °C

NANOG forward: 5′-GCTGAGATGCCTCACACGGAG-3′; 21 nt; 62% GC; Tm 71 °C

NANOG reverse: 5′-TCTGTTTCTTGACTGGGACCTTGTC-3′; 25 nt: 48%GC; Tm 69 °C Optimal Tm: 70 °C

GAPDH forward: 5′ATCTTCCAGGAGCGAGATCC-3′; 20 nt, 55% GC, Tm 66 °C

GAPDH reverse: 5′ACCACTGACACGTTGGCAGT-3′; 20 nt, 55% GC, Tm 70 °C

Optimal Tm: 61 °C

### 2.5. Proliferation Assays

DPSC isolates were plated in sterile, tissue culture-treated Corning Costar 96-well assay plates (Fisher Scientific 07-200-90; Fair Lawn, NJ, USA) at a concentration of 1.2 × 10^4^ cells/mL for three days. Experimental wells were treated with either 10 ng/mL of Gibco with recombinant vascular endothelial growth factor (VEGF, ThermoFisher #PHC9393; Fair Lawn, NJ, USA) or bone morphogenic protein (BMP-2, ThermoFisher #PHC7141; Fair Lawn, NJ, USA) or plated with 10 ug of MTA (Henry Schein #7040069; Melville, NY, USA). Plates were subsequently fixed with 10% neutral buffered formalin (ThermoScientific 22-045-400; Fair Lawn, NJ, USA) and stained using Gentian Violet 1% aqueous solution (Ricca Chemical 7647-01-0 from Fisher Scientific; Fair Lawn, NJ, USA). Absorbance was read using an ELx808 BioTek microplate reader (BioTek; Winooski, VT, USA) at 595 nm to calculate cellular proliferation and for comparison with negative controls.

### 2.6. Viability Assays

Cellular viability was determined using the Trypan Blue exclusion assay from Gibco (Fisher Scientific #15250061; Fair Lawn, NJ, USA). Cells (experimental, control) were processed using a BioRad TC20 automated cell counter (BioRad; Hercules, CA, USA) to determine the absolute and relative percentage of viable cells. Cell densities were also calculated for both experimental and negative controls.

### 2.7. Experimental Factors

Growth factors were obtained from Fisher Scientific, which included vascular endothelial growth factor (VEGF; catalog PHC9393) and bone morphogenic protein (BMP; catalog PHC7141). Experimental wells were treated with growth factor (VEGF or BMP-2) at physiologically relevant concentrations approximating 10 ng/mL—within the range of other studies of DPSC responsiveness to these growth factors [27,28,29,30]. Mineralized trioxide aggregate (MTA) was obtained from Henry Schein (catalog 7040069). In brief, MTA was mixed separately under sterile conditions in a BSL-2 biosafety cabinet according to the manufacturer instructions and 10 μL was transferred into each experimental well of a 96-well assay plate prior to cell plating (as described above).

### 2.8. Statistical Analysis

All parametric analyses of growth and viability were exported to Microsoft Excel (XLS) and subsequently analyzed using two-tailed t-tests. Statistical differences were calculated using an alpha level of 0.05 for statistical significance, as previously described [30,31]. Differences in RNA concentration were calculated based upon the doubling time (DT) or group, which are non-parametric or categorical groups, as previously described [28,29,30,31,32]. These data were analyzed using the Chi square test. Associations were estimated between growth or viability responsiveness (change) and DPSC categorical variables (rDT, iDT, sDT) using Pearson’s correlation coefficient or R^2^, as previously described [24,25,26,31,32].

## 3. Results

**Figure 1:**
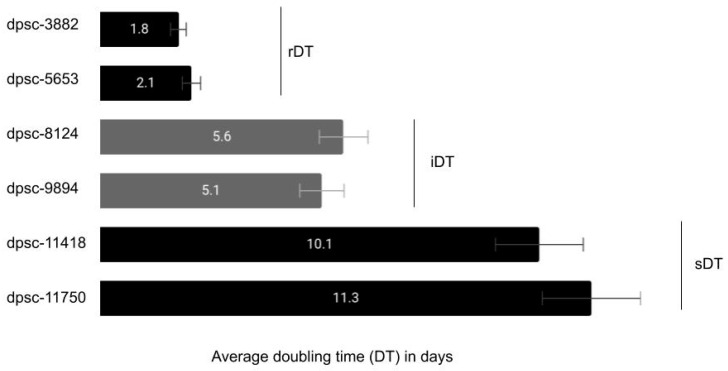
Cell culture reveals dental pulp stem cell (DPSC) doubling time (DT). Average doubling time for DPSC isolates was categorized as rapid or rDT (1–2 days; dpsc-3882, dpsc-5653), intermediate or iDT (5–6 days; dpsc-8124, dpsc-9894), or slow sDT (10–12 days; dpsc-11418, dpsc-11750), which remained consistent and were significantly different from one another, *p* = 0.0001.

Existing DPSC isolates were thawed and placed into culture with the average doubling time (DT) noted for each (Figure 1). These data demonstrated that two DPSC isolates exhibited a rapid and consistent doubling time or rDT of approximately two days (dpsc-3882, dpsc-5653). In addition, two DPSC isolates were identified as exhibiting an intermediate doubling time or iDT of approximately five to six days (dpsc-8124, dpsc-9894). Finally, two DPSC isolated were found to have slow doubling times or sDT between ten to twelve days (dpsc-11418, dpsc-11750). The average doubling times for each grouping (rDT, iDT, and sDT) were significantly different from each other, *p* = 0.0001.

RNA was subsequently extracted from each DPSC isolate and prepared for analysis (Table 1). These data revealed the average RNA concentration for all DPSC isolates was approximately 906 ng/μL. The average RNA concentration for the rDT DPSC isolates was 904.2 ng/μL, while the average RNA concentration for the iDT and sDT DPSC isolates was 904.2 and 919.4 ng/μL, respectively. These data revealed that no significant differences were observed between these groups, *p* = 0.325. The purity of the RNA was determined using the ratio of spectrophotometer absorbance readings at A260 and A280 nm, which revealed that average A260:A280 ratios for all DPSC isolates exceeded 1.65—the minimum acceptable standard for polymerase chain reaction (PCR) screening.

**Figure 2:**
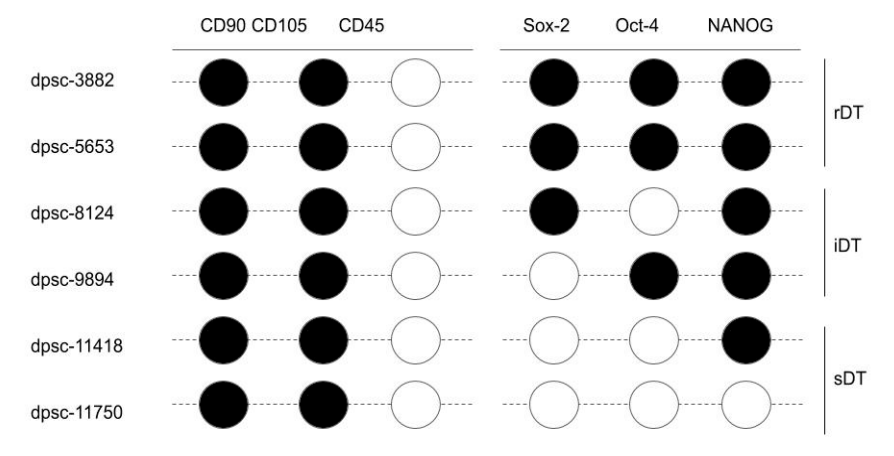
Results of DPSC isolate PCR biomarker screening. Positive results for CD90 and CD105 RNA expression were observed among all DPSC isolates, with negative results for CD45 expression. Differential results were observed among the DPSC isolates for Sox-2, Oct-4, and NANOG expression. Only the rDT (rapid) DPSC isolates expressed all three pluripotency biomarkers, with differential expression observed among the iDT and sDT DPSC isolates. Note: 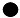 indicates RT-PCR band intensity above the limit of detection; 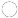 indicates an RT-PCR result below the limit of detection.

Screening of the RNA from each DPSC isolate for the ICST biomarkers, including CD45, CD90, and CD105, was performed (Figure 2). These data demonstrated that all DPSC isolates expressed both CD90 and CD105, but did not express CD45. In addition, the RNA was also screened for three additional mesenchymal stem cell pluripotency biomarkers, including Sox-2, Oct-4, and NANOG. These data revealed that both rapidly dividing DPSC isolates (rDT), dpsc-3882 and dpsc-5653, expressed all three biomarkers. The intermediate doubling time DPSC isolates (iDT), dpsc-8124 and dpsc-9894, both expressed NANOG but exhibited differential expression of Sox-2 and Oct-4. Finally, neither of the slow doubling time DPSC isolates (sDT) expressed Sox-2 or Oct-4 and only dpsc-11418 exhibited measurable NANOG expression.

**Figure 3:**
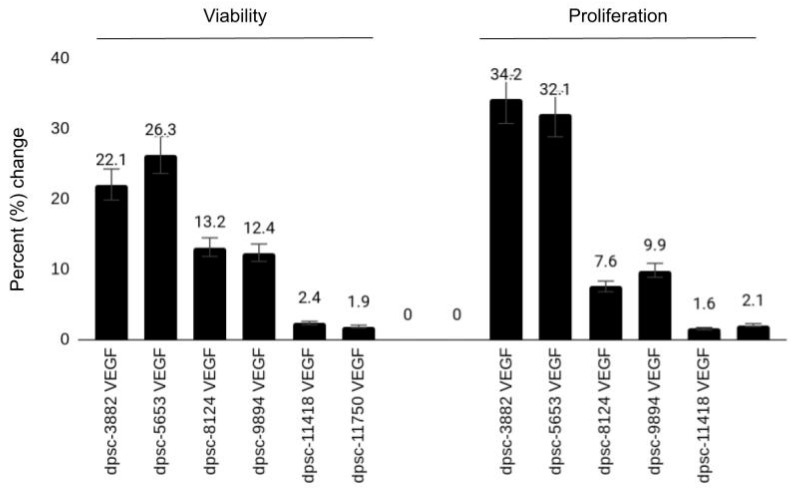
Effect of vascular endothelial growth factor (VEGF) on DPSC isolates. VEGF induced marked increases in viability and growth among the rDT (rapid) DPSC isolates (dpsc-3882, dpsc-5653), *p* = 0.0001. Moderate increases were observed among the iDT (intermediate) DPSC isolates (dpsc-8124, dpsc-9894), *p* = 0.002, with no significant differences observed among the sDT (slow) DPSC isolates (dpsc-11418, dpsc-11750), *p* = 0.441.

To determine the effects of VEGF on these DPSC isolates, all cells were plated with (experimental) and without (negative) VEGF at a concentration of 10 ng/mL—a physiologically relevant concentration used in other experimental protocols involving DPSC (Figure 3). These data demonstrated significant increases in cellular viability and proliferation among the rDT DPSC isolates (dpsc-3882: 22.1%, 34.2%, respectively; dpsc-5653: 26.3%, 32.1%, respectively), *p* = 0.0001. Although more moderate increases in both viability and growth were observed among the iDT DPSC isolates (dpsc-8124: 13.2%, 7.6%, respectively; dpsc-9894: 12.4%, 9.9%, respectively), these were significantly different from the negative (untreated) controls, *p* = 0.002. However, no significant differences in viability or growth were observed among the sDT DPSC isolates (dpsc-11418: 2.4%, 1.6%, respectively; dpsc-11750: 1.9%, 2.1%, respectively), *p* = 0.441. 

**Figure 4:**
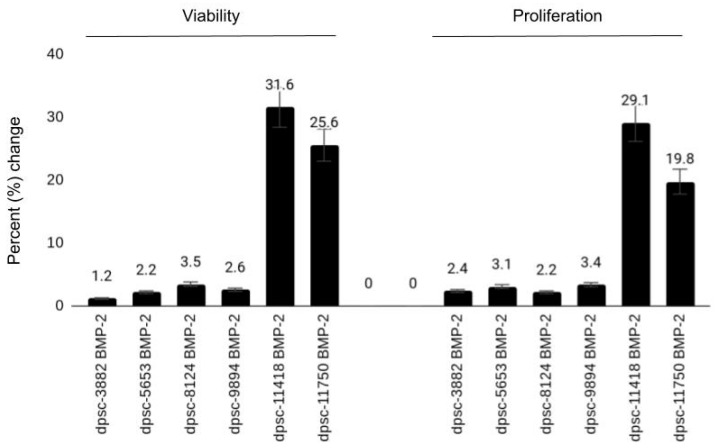
Effect of bone morphogenic protein (BMP-2) on DPSC isolates. BMP-2 did not alter viability or growth among the rDT (rapid) DPSC isolates (dpsc-3882, dpsc-5653), *p* = 0.541 with slight increases observed among the iDT (intermediate) DPSC isolates (dpsc-8124, dpsc-9894), *p* = 0.0811. However, BMP-2 induced significant increases among the sDT (slow) DPSC isolates (dpsc-11418, dpsc-11750), *p* = 0.0001.

To determine the effects of BMP-2 on these DPSC isolates, all cells were plated with (experimental) and without (negative) BMP-2 at a concentration of 10 ng/mL—a physiologically relevant concentration used in other experimental protocols involving DPSC (Figure 4). These results revealed no significant changes in either cellular viability or proliferation from the untreated controls among the rDT DPSC isolates (dpsc-3882: 1.2%, 2.4%, respectively; dpsc-5653: 2.2%, 3.1%, respectively), *p* = 0.541. Slight increases in both viability and growth were observed among the iDT DPSC isolates (dpsc-8124: 3.5%, 2.2%, respectively; dpsc-9894: 2.6%, 3.4%, respectively), although these were not significantly different from the negative (untreated) controls, *p* = 0.0811. However, significant differences in viability or growth were observed among the sDT DPSC isolates (dpsc-11418: 31.6%, 29.1%, respectively; dpsc-11750: 25.6%, 19.8%, respectively), *p* = 0.0001. 

**Figure 5:**
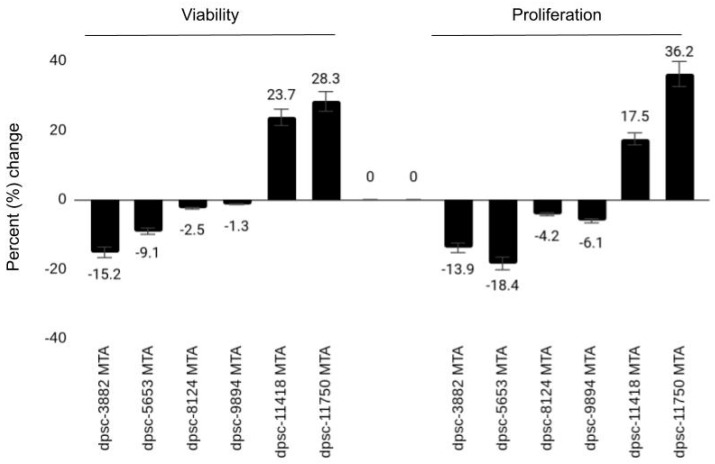
Effect of mineralized trioxide aggregate (MTA) on DPSC isolates. Both viability and growth were strongly inhibited by MTA among the rDT (rapid) DPSC isolates (dpsc-3882, dpsc-5653), *p* = 0.003. MTA also inhibited the iDT (intermediate) DPSC isolates (dpsc-8124, dpsc-9894) but to a lesser extent, *p* = 0.064. However, MTA induced significant increases among the sDT (slow) DPSC isolates (dpsc-11418, dpsc-11750), *p* = 0.0001.

Finally, each DPSC isolate was plated with and without the endodontic biomaterial mineralized trioxide aggregates (MTA) to explore any potential bioactivity of this dental material (Figure 5). A strong negative effect was observed between MTA administration and both the growth and viability of the rDT DPSC isolates (dpsc-3882: −15.2%, −13.9%, respectively; dpsc-5653: −9.1%, −18.4%, respectively), *p* = 0.003. A more moderate but still negative effect was observed among the iDT DPSC isolates (dpc-8124: −2.5%, −4.2%, respectively; dpsc-9894: −1.3%, −6.1%, respectively), *p* = 0.064. However, a significant positive effect was observed in viability and growth among the sDT DPSC isolates (dpsc-11418: 23.7%, 17.5%, respectively; dpsc-11750: 28.3%, 36.2%, respectively), *p* = 0.0001.

The responsiveness of the DPSC to each of the three experimental treatments, including the percentage change in growth and viability, was summarized (Table 2). These data demonstrated that a strong responsiveness to VEGF was observed among the rDT and iDT DPSC isolates, which correlated with the ICST and MSC biomarkers. For example, the rDT DPSC isolates (dpsc-3882, dpsc-5653) expressed all three MSC biomarkers (Sox-2, Oct-4, NANOG) and were the most responsive to VEGF administration. The iDT DPSC isolates expressing only two of the three MSC biomarkers (dpsc-8124; Sox-2, NANOG; dpsc-9894; Oct-4, NANOG) were less responsive to VEGF than either of the rDT DPSC isolates.

These correlations also suggested correlations with DPSC responses to MTA, which more strongly inhibited growth in proliferation among the rDT DPSC isolates (expressing all three MSC biomarkers) than the iDT DPSC isolates (expressing two MSC biomarkers). Interestingly, only the sDT DPSC isolates expressing one (NANOG) or none of the MSC pluripotency biomarkers exhibited positive responsiveness to MTA treatment. Similarly, the only group of DPSC isolates exhibiting responsiveness to BMP-2 treatment were the sDT DPSC isolates—which may suggest that the presence of two or more of the MSC pluripotency markers may be sufficient to inhibit DPSC responsiveness.

To evaluate the association between the responsiveness of DPSC isolates (either growth or viability) and DPSC category (rDT, iDT, sDT), Pearson’s correlation was performed, which demonstrated strong associations may exist. For example, the association between DPSC isolate responsiveness in viability to VEGF administration was R^2^ = 0.868, while the association between responsiveness in growth to VEGF was R^2^ = 0.82. Similarly, the responsiveness in viability and growth of the DPSC isolates by category to BMP-2 was R^2^ = 0.667 and R^2^ = 0.588, respectively. Finally, the correlations between MTA and DPSC responsiveness were R^2^ = 0.904, R^2^ = 0.831.

## 4. Discussion

The primary objective of this study was to understand the biology and biotechnology needed to more effectively modulate DPSC responsiveness and phenotypes, using BMP, VEGF, and MTA. The results of this study have demonstrated that specific biological determinants of DPSC pluripotency, including Sox-2, Oct-4, and NANOG, may be effective biomarkers to determine the responsiveness of DPSC isolates to various stimuli—at least in vitro [31,32]. These results support other studies of DPSC characteristics and biomarkers that evaluate the potential for reprogramming and differentiation [33,34].

In addition, these data also confirm previous studies of DPSC responsiveness to various growth factors, including VEGF and BMP-2 [18,19,20,35,36]. One of these previous studies evaluated the pluripotency transcription factors Sox-2 and Klf-4, but did not evaluate the potential responsiveness of DPSC isolates to any stimulus or growth factors based upon expression of these markers [33]. However, this study may be among the first to demonstrate the differential responsiveness of DPSC to these growth factors based upon the combination of these specific pluripotency biomarkers (Sox-2, Oct-4, NANOG) and growth characteristics, such as doubling time or proliferation.

Studies from other groups have identified similar biomarkers among DPSC that may be used to determine functional differentiation capabilities, such as the expression of Oct-4, Sox-2, and Klf-4 with Lin28, which may determine DPSC isolates capable of odontoblastic differentiation [37]. Other research groups have focused on the identification of additional growth factors that may promote specific and directed differentiation of DPSC isolates, such as basic fibroblast growth factor (bFGF) promotion of DPSC neural differentiation and fibroblast growth factor (FGF-2) promotion of DPSC odontoblast differentiation [38,39].

Although the results of this study are novel and suggest effective methods for evaluating and selecting differentiation potential among DPSC isolates, there are several limitations associated with this study that must be considered. For example, any association between the expression of these biomarkers and DPSC responsiveness may be coincidental and not causative. The retrospective nature of this study, combined with both financial and technical barriers that limited the ability of this group to evaluate this possibility through knockout or silencing RNA, suggest that future studies may need to include methods to rule out these possibilities.

In addition, this study was limited to a small number of DPSC isolates. Expanding this study to include more DPSC isolates with variable expression of these pluripotency biomarkers will provide substantial information that could validate the findings of the current study. In addition, a more comprehensive evaluation of other potential biomarkers, such as non-coding microRNA, may provide more specific and targeted methods for bioengineering and biotechnology applications utilizing DPSC [40,41,42].

## 5. Conclusions

These data suggest that some DPSC isolates (but not all) are capable of a robust and significant in vitro response to stimuli, although this response is not universal. Although some biomarkers and phenotypes that distinguish and characterize these DPSC isolates may facilitate the ability to predict phenotypic responses and changes in growth or viability potential, more research is needed to determine the other intrinsic and extrinsic factors that may contribute to and modulate these DPSC responses for biotechnology and bioengineering applications.

## Figures and Tables

**Table 1:** RNA concentration from DPSC isolates.

DPSC Isolate	RNA Quantification	Statistical Analysis	RNA Purity
dpsc-3882	921.1 ng/μL	–	1.74
dpsc-5653	887.3 ng/μL	–	1.77
dpsc-8124	925.6 ng/μL	–	1.72
dpsc-9894	913.2 ng/μL	–	1.65
dpsc-11418	879.4 ng/μL	–	1.91
dpsc-11750	910.1 ng/μL	–	1.83
rDT	904.2 ng/μL	*X*^2^ = 0.325	ave. = 1.75
iDT	919.4 ng/μL	d.f. = 2	ave. = 1.69
sDT	894.75 ng/μL	*p* = 0.8502	ave. = 1.87

**Table 2:** Responsiveness of DPSC isolates to stimulus.

DPSC Isolate	VEGF (Viability, Growth)	BMP-2 (Viability, Growth)	MTA (Viability, Growth)
dpsc-3882	22.1%, 34.2%	1.2%, 2.4%	−15.2%, −13.9%
dpsc-5653	26.3%, 32.1%	2.2%, 3.1%	−9.1%, −18.4%
dpsc-8124	13.2%, 7.6%	3.5%, 2.2%	−2.5%, −4.2%
dpsc-9894	12.4%, 9.9%	2.6%, 3.4%	−1.3%, −6.1%
dpsc-11418	2.4%, 1.6%	31.6%, 29.1%	23.7%, 17.5%
dpsc-11750	1.9%, 2.1%	25.6%, 19.8%	28.3%, 36.2%
rDT	++, +++	+, +	−−, −−
iDT	++, +	+, +	−, −
sDT	+, +	++, ++	++, ++
Correlation (R^2^)	R^2^ = 0.868, R^2^ = 0.82	R^2^ = 0.667, R^2^ = 0.588	R^2^ = 0.904, R^2^ = 0.831

## Data Availability

The data presented in this study are available on request from the corresponding author. The data are not publicly available due to the study protocol data protection parameters.

## Abbreviations

**Abbreviation**	**Name**
DPSC	Dental pulp stem cell
VEGF	vascular endothelial growth factor
BMP	bone morphogenic protein
MTA	mineralized trioxide aggregates
SHED	stem cells from human exfoliated deciduous teeth
SCAP	stem cells from the apical papilla
UNLV	University of Nevada, Las Vegas
OPRS	Office for the Protection of Research Subjects
SDM	School of Dental Medicine
DMSO	dimethyl sulfoxide
DMEM	Dulbecco’s Modified Eagle’s Medium
FBS	fetal bovine serum
rDT	rapid doubling time
iDT	intermediate doubling time
sDT	slow doubling time
PCR	polymerase chain reaction
ISCT	International Society for Cellular Therapy
MSC	mesenchymal stem cell
GAPDH	glyceraldehyde 3-phosphate dehydrogenase
bFGF	basic fibroblast growth factor
FGF	fibroblast growth factor
